# AtHKT1;1 Mediates Nernstian Sodium Channel Transport Properties in *Arabidopsis* Root Stelar Cells

**DOI:** 10.1371/journal.pone.0024725

**Published:** 2011-09-09

**Authors:** Shaowu Xue, Xuan Yao, Wei Luo, Deepa Jha, Mark Tester, Tomoaki Horie, Julian I. Schroeder

**Affiliations:** 1 Division of Biological Sciences, Cell and Developmental Biology Section, University of California, San Diego, La Jolla, California, United States of America; 2 Australian Centre for Plant Functional Genomics, University of Adelaide, South Australia, Australia; 3 State Key Laboratory of Crop Genetics and Germplasm Enhancement, College of Resources and Environmental Sciences, Nanjing Agricultural University, Nanjing, China; United States Department of Agriculture, Agricultural Research Service, United States of America

## Abstract

The *Arabidopsis* AtHKT1;1 protein was identified as a sodium (Na^+^) transporter by heterologous expression in *Xenopus laevis* oocytes and *Saccharomyces cerevisiae*. However, direct comparative *in vivo* electrophysiological analyses of a plant HKT transporter in wild-type and *hkt* loss-of-function mutants has not yet been reported and it has been recently argued that heterologous expression systems may alter properties of plant transporters, including HKT transporters. In this report, we analyze several key functions of AtHKT1;1-mediated ion currents in their native root stelar cells, including Na^+^ and K^+^ conductances, AtHKT1;1-mediated outward currents, and shifts in reversal potentials in the presence of defined intracellular and extracellular salt concentrations. Enhancer trap *Arabidopsis* plants with GFP-labeled root stelar cells were used to investigate AtHKT1;1-dependent ion transport properties using patch clamp electrophysiology in wild-type and *athkt1;1* mutant plants. AtHKT1;1-dependent currents were carried by sodium ions and these currents were not observed in *athkt1;1* mutant stelar cells. However, K^+^ currents in wild-type and *athkt1;1* root stelar cell protoplasts were indistinguishable correlating with the Na^+^ over K^+^ selectivity of AtHKT1;1-mediated transport. Moreover, AtHKT1;1-mediated currents did not show a strong voltage dependence *in vivo*. Unexpectedly, removal of extracellular Na^+^ caused a reduction in AtHKT1;1-mediated outward currents in Columbia root stelar cells and *Xenopus* oocytes, indicating a role for external Na^+^ in regulation of AtHKT1;1 activity. Shifting the NaCl gradient in root stelar cells showed a Nernstian shift in the reversal potential providing biophysical evidence for the model that AtHKT1;1 mediates passive Na^+^ channel transport properties.

## Introduction

Glycophytic plants are sensitive to high concentrations of sodium chloride (NaCl) salt in soils [1]. Salt accumulates in crop lands due to irrigation and due to natural NaCl occurrence or deposition, with over 40% of irrigated croplands being negatively affected by salinity stress [2]. Over-accumulation of sodium (Na^+^) ions in plants is the major contributor to salinity stress [3], [4], [5], [6]. Na^+^ transporters have key diverse functions in protecting plants from salinity stress [5], [6], including Na^+^ sequestration into plant vacuoles [7], Na^+^ extrusion from cells at the plasma membrane via Na^+^/H^+^ antiport [8], and avoiding Na^+^ over-accumulation in leaves [9].

The *Arabidopsis* genome includes only one *HKT* transporter gene, *AtHKT1;1* that is highly expressed in roots and moderately expressed in shoots [10]. AtHKT1;1 was shown to encode a Na^+^ transporter in yeast and *Xenopus laevis* oocytes [10]. Chimera analyses of AtHKT1;1 and the Na^+^/K^+^ transporting wheat HKT1 (TaHKT2;1) transporter identified an important selectivity filter serine residue that functions in the preferential Na^+^ selectivity of AtHKT1;1 in these heterologous systems [11].

The question of why plants express Na^+^ selective HKT transporters was revealed through genetic analyses. AtHKT1;1 was shown to protect leaves from Na^+^ over-accumulation by reducing Na^+^ levels in leaves, while also maintaining higher concentrations of Na^+^ in *Arabidopsis* roots [9]. Many K^+^-binding proteins, protein synthesis [4] and unknown aspects of photosynthetic metabolism in leaves are particularly sensitive to sodium over-accumulation. Thus maintaining low levels of Na^+^ in leaves (leaf Na^+^ exclusion) is an important strategy for salinity resistance in crops [6], [12], [13], [14]. *athkt1;1* knock-out mutant plants showed over-accumulation of Na^+^ in leaves, resulting in leaf chlorosis, and concomitant under-accumulation of Na^+^ in roots [9]. AtHKT1;1 expression was found in the vasculature, together suggesting that AtHKT1;1 functions in long distance root/shoot Na^+^ transport and leaf Na^+^ exclusion [9], [15]. Forward genetic screens of leaf Na^+^ over-accumulation mutants isolated *athkt1;1* mutant alleles, further strengthening the model that AtHKT1;1 functions in leaf Na^+^ exclusion [15], [16].

Immuno-histochemical localization using an AtHKT1;1 antibody detected AtHKT1;1 protein in the plasma membrane of *Arabidopsis* xylem parenchyma cells and *AtHKT1;1* promoter β-glucuronidase (GUS) analyses corresponded with this finding [17]. Furthermore, xylem sap analyses demonstrated that AtHKT1;1 reduces xylem sap Na^+^ concentrations in wild-type plants exposed to salinity stress [17], [18]. These findings led to the present model for AtHKT1;1 function, in which AtHKT1;1 removes Na^+^ from the xylem sap in response to salinity stress, thus mediating leaf Na^+^ exclusion [17]. Phloem loading of Na^+^ via AtHKT1;1 was initially proposed as the mechanism by which leaf Na^+^ exclusion is mediated [15]. However, this model has been questioned, based on the preferential xylem parenchyma localization of AtHKT1;1 protein [17] and unidirectional Na^+^ tracer flux data suggesting no function of AtHKT1;1 in recirculating Na^+^ to roots [19].

Independent research analyzing the quantitative trait locus (QTL), *SKC1*, in rice, which shows enhanced salinity resistance caused by increased K^+^ accumulation in leaves showed that the *SKC1* locus encodes a close rice homolog to *AtHKT1;1*, *OsHKT1;5* [20]. This research led to the same model for the function of the rice orthologue OsHKT1;5 in mediating Na^+^ exclusion in leaves via Na^+^ removal from the xylem sap, which stimulates K^+^ loading into the xylem vessel, resulting in increased K^+^ accumulations in shoots [20]. A class 1 *Mesembryanthemum crystallinum* HKT transporter, McHKT1;1, is targeted to the plasma membrane of xylem parenchyma cells [21], indicating that the leaf Na^+^ exclusion function of class 1 HKT transporters may occur in halophytic plants as well.

Further analyses of *athkt1;1* mutants, including tracer flux analyses and natural variants in *AtHKT1;1* also showed the function of AtHKT1;1 in mediating Na^+^ removal from the xylem sap during salinity stress as a mechanism mediating salinity tolerance [19], [22]. Moreover, over-expression of AtHKT1;1 in root stele cells using enhancer trap expression resulted in increased salinity resistance, increased Na^+^ current activities in stelar cells and enhanced leaf Na^+^ exclusion in transgenic *Arabidopsis* lines further illustrating the potential of AtHKT1;1 in engineering salinity resistance [23]. A recent study showed that cytokinin and type-B response regulators ARR1 and ARR12 regulate expression of *AtHKT1;1* [24].

A distinct type of Na^+^ selective HKT transporter, OsHKT2;1 [25], belongs to a different class “2” of HKT transporters. Class 2 HKT transporters analyzed thus far have distinct functions from the above class 1 HKT transporters. OsHKT2;1 mediates Na^+^ influx into roots when rice roots are K^+^ starved [26]. Several class 2 HKT transporter transcripts are induced in roots by K^+^ starvation [27], [28]. Other K^+^ uptake channels and transporters are also induced by K^+^ starvation [29], [30], [31], [32], [33], [34]. OsHKT2;1 mediates uptake of Na^+^ into K^+^-starved rice roots enabling Na^+^ to function as an alternate osmoticum to K^+^ ions. Upon salinity stress, however, the large rates of OsHKT2;1-mediated root Na^+^ influx are rapidly down-regulated, thus protecting roots from Na^+^ over-accumulation [26]. Recent research has demonstrated Ca^2+^ and Mg^2+^ permeabilities of a rice class 2 HKT transporter in *Xenopus* oocytes [35], [36], and K^+^ competitively inhibits these Ca^2+^ and Mg^2+^ permeabilities [36], further highlighting the need to characterize HKT transporter selectivity *in planta*, as pursued in the present study.

Major salinity tolerance quantitative trait loci (QTL) in wheat, *Kna1*, *NAX1* and *NAX2*, have been isolated and characterized [37], [38], [39], [40]. Furthermore, these QTL in wheat control Na^+^ levels in the xylem sap and the leaf base (sheath) and protect leaf blades from Na^+^ over-accumulation [41], [42]. All three of these QTL exhibit polymorphisms in copies of class-1 *HKT* genes within the mapping regions of these three QTL, indicating that *AtHKT1;1*-related genes and mechanisms may be responsible for Na^+^ tolerance in these wheat lines [43], [44] and that these wheat HKT proteins may share analogous or similar functions with the *Arabidopsis* AtHKT1;1 transporter.

Previous studies have suggested that HKT transporters show ion channel-like functions [11], [35], [45], [46], [47], [48]. The transmembrane topology of HKT transporters has revealed 8 transmembrane domains with 4 selectivity filter containing pore loops [48], [49], consistent with selectivity pore mutagenesis studies [11], [50]. However, an important missing link towards investigating this hypothesis is that the reversal potentials of HKT transporters have not yet been investigated under defined and experimentally shifted conditions in which the cytoplasmic ion concentration is clamped, as can be analyzed using patch clamp electrophysiological analyses. Furthermore, reversal potential shifts of HKT transporters have not yet been analyzed in electrophysiological studies in their native plant cells. Although, HKT transporters are arguably the best-characterized Na^+^-permeable transporters in plants [12], direct functional electrophysiological recordings of HKT transporters in their native plant cells has only been reported once, demonstrating increased current magnitudes in HKT-over-expressing stele cells compared to wild-type cells [23]. Moreover, a study analyzing 5′ UTR modified HKT transporter constructs and expression in yeast led to the hypothesis that these heterologous systems do not reflect the ion selectivity properties of plant HKT transporters *in planta* [51] (but see: [47]). To investigate this important hypothesis, HKT transporter function thus needs to be addressed in native plant membranes by direct electrophysiological investigations of wild-type and loss-of-function mutations.

In the present study we used enhancer trap lines [23], [52], [53] that GFP label root stele cells to investigate ionic currents in wild-type and *AtHKT1;1* knock-out plants. Direct patch clamping allowed us to address several key questions on the functional and biophysical properties of an HKT transporter in its native cells, including:

1. Can AtHKT1;1 transport large rates of Na^+^ and/or K^+^ *in vivo*?

2. AtHKT1;1 in *Arabidopsis* and OsHKT1;5 in rice, not only reduce Na^+^ accumulation in the xylem sap and leaves, but also increase K^+^ concentrations in xylem sap and leaves [17], [20]. Does AtHKT1;1 mediate K^+^ efflux from native cells, or is K^+^ efflux activity mediated by an AtHKT1;1 independent mechanism, such as outward-rectifying K^+^ channels [54], [55], [56], [57], [58]?

3. Can AtHKT1;1 transport both inward (into the cell) and outward Na^+^ flux in their native cells?

4. Do AtHKT1;1-mediated currents show Nernstian reversal potential changes when defined ion gradients are shifted indicative of passive channel transport activity?

## Results

*AtHKT1;1* is more highly expressed in roots than shoots [10] and *AtHKT1;1* transcript is induced by salinity stress [17]. HKT1;1 promoter fusions with the β-glucuronidase (GUS) reporter gene as well as immuno-localization have shown that *AtHKT1;1* is expressed in the vasculature [9] in xylem parenchyma cells [17].

**Figure 1:**
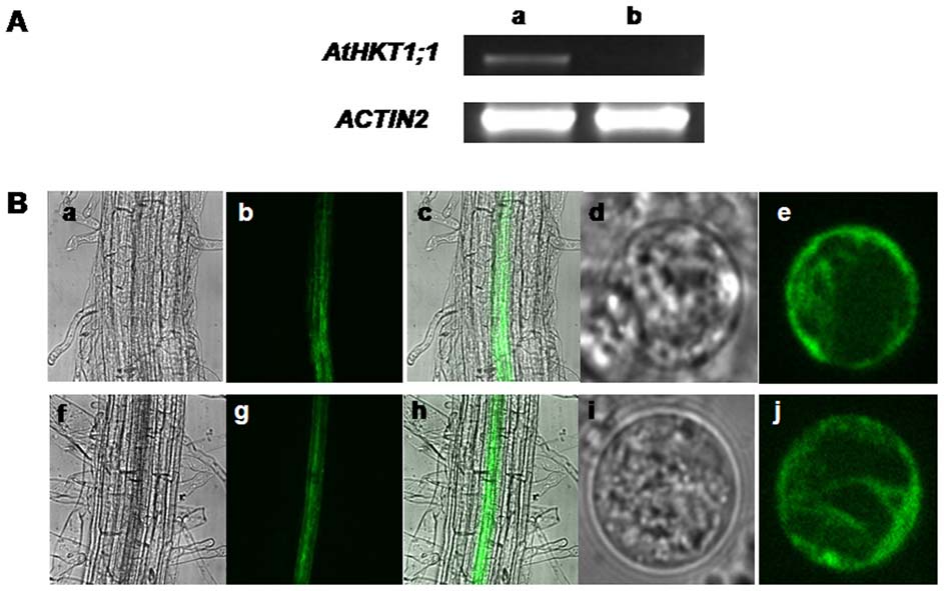
Fluorescence labeling of root stelar cells in enhancer trap (E2586) plants. **A**) RT-PCR analysis of expression level of *AtHKT1;1* gene in E2586 (a) and *athkt1;1-4* X E2586 (b) plants. **B**) GFP-marked stele in root (a–c, f–h) and root stelar cell protoplasts (d, e, i and j). E2586 (a–e) and *athkt1;1-4* X E2586 (f–j). (a, d, f, i): bright field; (b, e, g, j): fluorescence images; (c): merged graphs (a) and (b); (h): merged graphs (f) and (g).

To directly characterize AtHKT1;1 function in root stelar cells, E2586 enhancer trap plants were used, which exhibit cell type-specific GFP expression in the root stele [23]. Homozygous plants were generated by crossing the *athkt1;1-4* T-DNA disruption line (Figure 1A) with E2586 plants. The stelar cell-specific GFP expression of the E2586 line was confirmed (Figure 1B a–e). *athkt1;1-4* X E2586 plants also showed stable GFP fluorescence in root steles (Figure 1B f–j). RT-PCR analysis confirmed that *AtHKT1;1* is expressed in E2586 plants, while not in *athkt1;1-4* X E2586 plants (Figure 1A). Root protoplasts were isolated and protoplasts showing GFP fluorescence could be readily identified (Figure 1B e, j).

**Figure 2:**
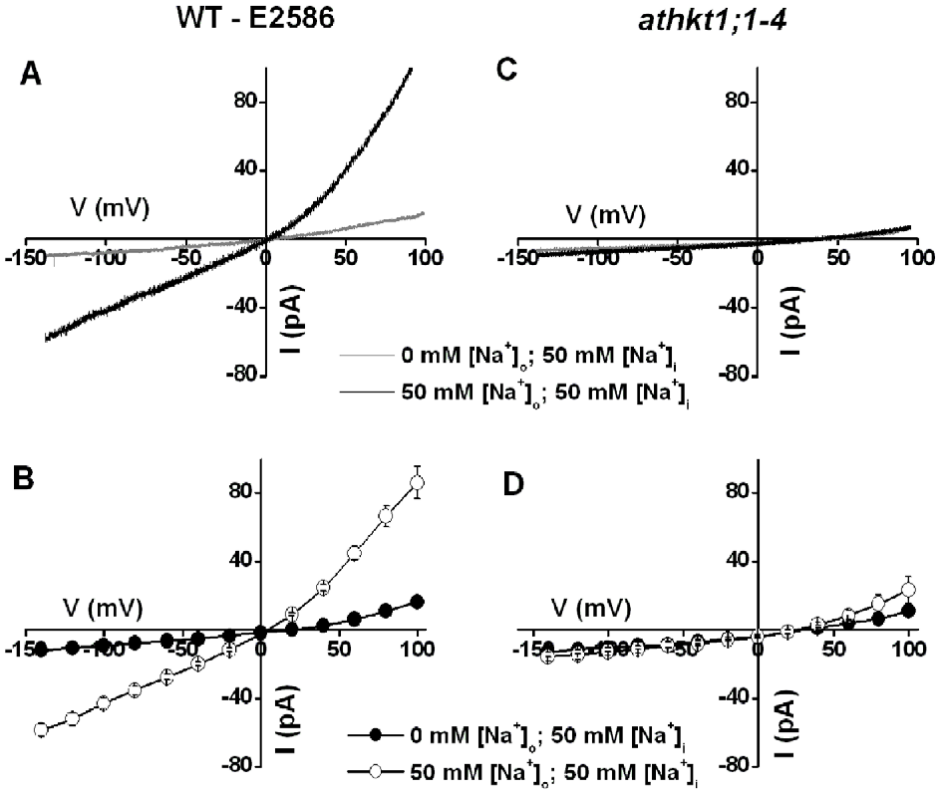
HKT1;1-mediated Na^+^ currents in E2586 wild-type (WT) and lack thereof in *athkt1;1-4* GFP-labeled root stelar cell protoplasts. **A**) Typical whole-cell currents activated by rapid voltage ramps (5 mV ms^−1^, +99 to −141 mV) with the bath solution containing 0 (grey trace) or 50 mM NaCl (black trace) in wild-type E2586 root stelar cell protoplasts. **B**) Average current-voltage relationships recorded as in (A) from five wild-type E2586 protoplasts. Filled circles and open circles represent 0 mM and 50 mM NaCl in the bath respectively. The data are mean ± SE. The average current of −59 pA activated at a voltage of −140 mV corresponds to a current density of −35 mA m^−2^ with an average protoplast diameter of 23 µm. **C**) Typical whole-cell recordings using the same voltage ramps as in (A) with the bath solution containing 0 (grey trace) or 50 mM NaCl (black trace) in *athkt1;1-4* loss-of-function mutant root stelar cell protoplasts. **D**) Average current-voltage relationships recorded as in (C) from five *athkt1;1-4* mutant protoplasts. Filled circles and open circles represent 0 mM and 50 mM NaCl in the bath respectively. The data are mean ± SE. The bath solution contained 0/50 mM NaCl, 2 mM CaCl_2_, 5 mM MES, pH 5.7 adjusted with Tris. The pipette solution contained 50 mM NaCl, 1.3 mM CaCl_2_, 3 mM EGTA, 5 mM HEPES, pH 7.2 adjusted with Tris. The osmolalities of bath and pipette solutions were adjusted to 290–300 mmol·Kg^−1^ with D-mannitol. Liquid junction potential (LJP) was +0.3 mV for 50 mM [Na^+^]_o_ and −12 mV for 0 mM [Na^+^]_o_ (calculated by Clampex 10) and was corrected in (A–D).

We analyzed whether ionic currents could be resolved in GFP-labeled cells with high NaCl concentrations on both sides of the membrane in whole-cell patch-clamp recordings. Whole-cell currents were examined in GFP-labeled root stelar cell protoplasts of E2586 (from here on referred to as wild-type) as well as *athkt1;1-4* plants. Upon application of one second voltage ramps (from +99 mV to −141 mV), whole-cell ion currents were observed in wild-type root stelar cells. These whole-cell currents were largely voltage-independent with a slightly increased slope (rectification) at positive voltages (Figure 2A and B). These ionic current were dependent on the presence of [Na^+^] in the bath solution, with large currents resolved with 50 mM NaCl in the bath and pipette solutions (Figure 2A and B). When NaCl was removed from the bath solution, these large whole-cell currents vanished, including the large outward currents, indicating an unexpected additional role for extracellular Na^+^ in gating of AtHKT1;1 (Figure 2A and B).

When the same voltage ramps were applied in *athkt1;1-4* root stelar cells, no significant currents were observed, even with 50 mM NaCl on both sides of the membrane (Figure 2C and D). These data indicated that Na^+^ and/or Cl^−^ currents vanished in *athkt1;1* knock-out mutant stelar cells (Figure 2C and D).

**Figure 3:**
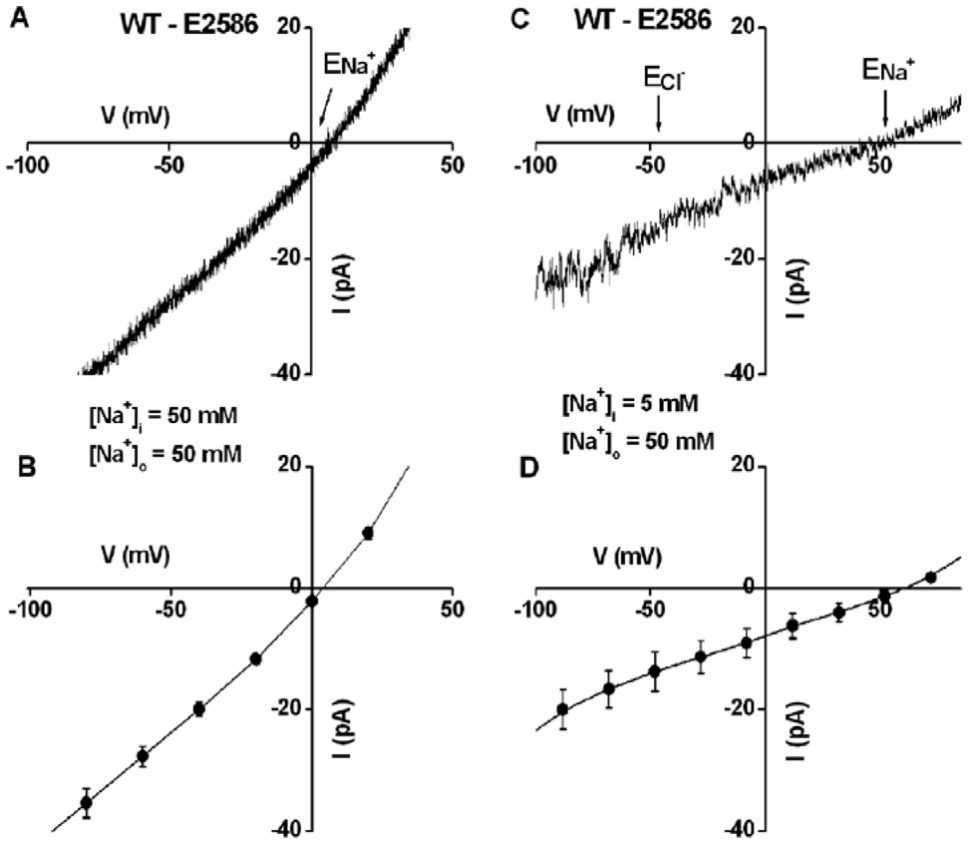
AtHKT1;1 mediates Na^+^ currents. **A**) Whole-cell current recorded with 50 mM NaCl in both the bath and pipette solutions in wild-type root stelar cells. **B**) Average current-voltage relationships recorded as in (A) from five protoplasts. Liquid junction potential (LJP) in (A and B) was +0.3 mV (calculated by Clampex 10) and was corrected in (A) and (B). **C**) Whole-cell current recorded with 5 mM NaCl in the pipette solution and 50 mM NaCl in the bath solution. **D**) Average current-voltage relationships recorded as in (C) from five protoplasts. LJP in (C and D) was +8 mV (calculated by Clampex 10) and was corrected in (C) and (D). E_Na+_: Na^+^ equilibrium potential. E_Cl_^−^: Cl^−^ equilibrium potential. The data in (B and D) are mean ± SE. The average currents of −19 and −35 pA activated at a voltage of −80 mV correspond to current densities of −11 and −21 mA m^−2^ with an average protoplast diameter of 23 µm. The bath solution in (A) to (D) contained 50 mM NaCl, 2 mM CaCl_2_, 5 mM MES, pH 5.7 adjusted with Tris. The pipette solution in (A) and (B) contained 50 mM NaCl, 1.3 mM CaCl_2_, 3 mM EGTA, 5 mM HEPES, pH 7.2 adjusted with Tris. The pipette solution in (C) and (D) contained 5 mM NaCl, 1.3 mM CaCl_2_, 3 mM EGTA, 5 mM HEPES, pH 7.2 adjusted with Tris. The osmolalities of bath and pipette solutions were adjusted to 290–300 mmol·Kg^−1^ with D-mannitol.

To determine the ion selectivity of AtHKT1;1-mediated currents, reversal potentials of whole-cell currents were analyzed in stelar cells of wild-type plants. With symmetrical 50 mM NaCl in the bath and pipette solutions, the reversal potential was 3.8±1.2 mV (n = 5), which was close to the equilibrium potential for Na^+^ (E_Na_^+^ = 0 mV) and Cl^−^ (E_Cl_^−^  =  −0.7 mV) (Figure 3A and B). With 50 mM NaCl in the bath solution and 5 mM NaCl in the pipette solution, the reversal potential shifted to +53.1±4.7 mV (n = 5), which was close to E_Na_^+^ of +56.1 mV and substantially different from E_Cl_^−^ of −46.3 mV (corrected for ionic activity) (Figure 3C and D). These data show that AtHKT1;1 mediates a Na^+^ current in root stelar cells. Furthermore, AtHKT1;1-mediated Na^+^ currents showed a Nernstian reversal potential change upon shifting the [Na^+^] gradient.

**Figure 4:**
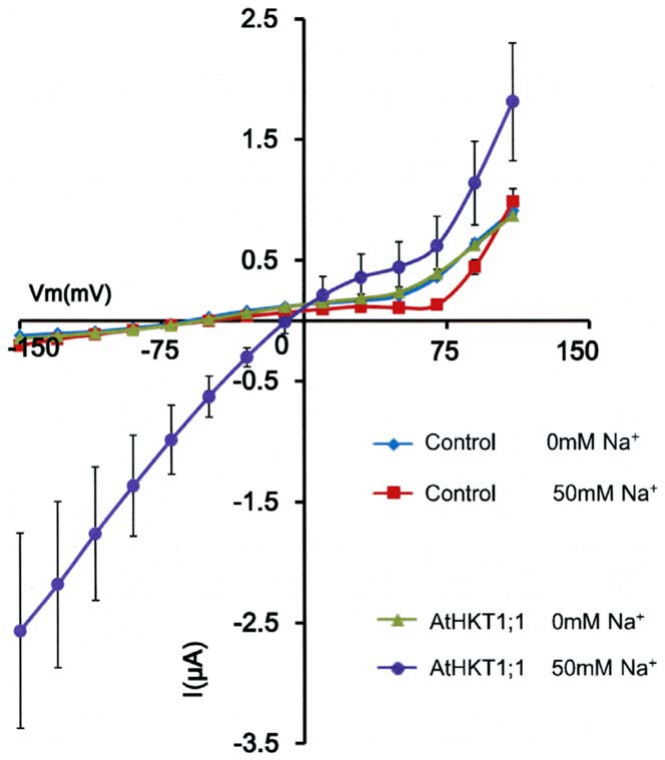
Removal of extracellular Na^+^ in AtHKT1;1 expressing *Xenopus* oocytes reduces AtHKT1;1-mediated outward currents. Recordings were carried out 1 to 2 days after *AtHKT1;1* cRNA injection (source: Columbia ecotype). Oocytes were clamped in perfusion solutions containing either 0 mM NaCl or 50 mM NaCl. Applied membrane potentials ranged from +110 to −150 mV. Mean steady-state currents (±SEM) recorded in oocytes injected with either 20 ng of Col-0 wild-type *AtHKT1;1* (n = 8) cRNA, or with H_2_O (n = 13) as control.

To investigate whether the strong reduction in wild-type Na^+^ currents upon removing extracellular Na^+^ ions in root stelar cells (Figure 2A and B) is due to a direct gating (external Na^+^ sensing) function of AtHKT1;1, we analyzed AtHKT1;1-mediated currents in *Xenopus* oocytes. The Columbia *AtHKT1;1* cDNA was isolated for this purpose to match the ecotype of the present patch clamp studies in native root stelar cells. Note that the previously described *AtHKT1;1* cDNA was isolated from the *Landsberg erecta* ecotype [10]. Removal of extracellular Na^+^ bathing oocytes reduced AtHKT1;1-mediated outward Na^+^ currents, even though the electrochemical driving force for Na^+^ efflux was greatly enhanced under these conditions (Figure 4). Experiments in *Xenopus* oocytes indicated that endogenous outward currents remained in the control and AtHKT1;1-expressing oocytes when recordings were conducted in 0 mM bath Na^+^ solution (Figure 4).

**Figure 5:**
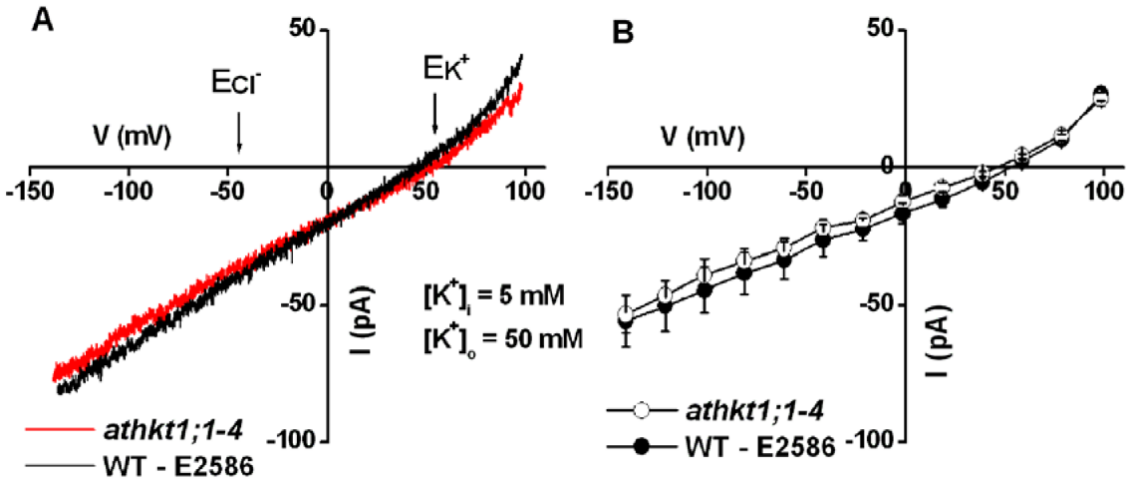
Potassium currents in wild-type (WT-E2586) and *athkt1;1-4* root stelar cell protoplasts are indistinguishable. **A**) Whole-cell currents activated by voltage ramps (5 mVms^−1^, +99 to −141 mV) with 5 mM KCl in the pipette solution and 50 mM KCl in the bath solution in wild-type (black trace) and *athkt1;1-4* mutant (red trace) root stelar cells. **B**) Average current-voltage relationships recorded as in (A) from five *athkt1;1-4* mutant protoplasts (open circles), five WT (E2586) protoplasts (filled circles). Average currents of −53 and −56 pA activated at a voltage of −141 mV correspond to current densities of −32 and −34 mA m^−2^ with an average protoplast diameter of 23 µm. The bath solution contained 50 mM KCl, 2 mM CaCl_2_, 5 mM MES, pH 5.7 adjusted with Tris. The pipette solution contained 5 mM KCl, 1.3 mM CaCl_2_, 3 mM EGTA, 5 mM HEPES, pH 7.2 adjusted with Tris. The osmolalities of bath and pipette solutions were adjusted to 290–300 mmol·Kg^−1^ with D-mannitol. Liquid junction potential (LJP) in (A and B) was +0.7 mV (calculated by Clampex 10) and was corrected in (A) and (B).

Next, the potassium conductance of AtHKT1;1-dependent currents in root stelar cells was investigated in wild-type and *athkt1;1-4* mutant plants. Figure 5A shows that large currents were activated by voltage ramps in both wild-type and *athkt1;1-4* plants. The bath solution contained 50 mM KCl and the pipette solution which dialyzes the cytosol contained 5 mM KCl. The reversal potentials of whole-cell currents were +52.2±2.7 mV for wild-type and +47.2±1.2 mV for *athkt1;1-4* mutant cells (Figure 5B), which were close to the equilibrium potential for K^+^ (E_K_^+^  =  +54.9 mV) and substantially different from the chloride equilibrium potential (E_Cl_^−^) of -44.3 mV. Thus the currents in KCl solutions in root stelar cells of wild-type and *athkt1;1-4* mutant plants (Figure 5) were mediated by K^+^ ions. The reversal potentials of currents for wild-type and *athkt1;1-4* mutants showed no significant difference (*P* = 0.15). Furthermore, the amplitude of the currents showed no significant differences among wild-type and *athkt1;1-4* knock-out mutant plants. At a voltage of -141 mV, the K^+^ current was −56±9 pA (−34±5 mA m^-2^; n = 5) for wild-type and −53±7 pA (−32±4 mA m^−2^; n = 5) for *athkt1;1-4* plants (*P*>0.05, student's t-test).

## Discussion

AtHKT1;1 was characterized as a Na^+^ transporter in yeast and *Xenopus laevis* oocytes [10]. In the present report, we have directly analyzed ionic currents mediated by AtHKT1;1 in GFP-labeled in *Arabidopsis* root stelar cells. A recent study showed that AtHKT1;1 over-expression in root stelar cells increased the amplitude of Na^+^ currents in these cells [23]. In the present study, several key questions and functional properties of AtHKT1;1 have been addressed, including:

1. Do the properties of AtHKT1;1 differ from those characterized in heterologous expression systems?

2. Analysis of the Na^+^ vs. K^+^ transport ability of AtHKT1;1 *in vivo*,

3. Patch clamp comparison of knock-out and wild-type root stelar cells allowed analysis of the question whether AtHKT1;1 can carry outward Na^+^ currents.

4. Do AtHKT1;1-mediated currents exhibit Nernstian shifts in reversal potentials, typical of passive ion channels, with a shifting ionic gradient when both the cytoplasmic and extracellular solutions are defined?

A previous study has suggested that expression of plant HKT transporters in heterologous cells changes the ion selectivity of these transporters [51], although more recent research from the same group has also pointed to possible limitations in transporter mRNA expression with the constructs used to derive this conclusion [59]. None-the-less, this important hypothesis needed to be investigated. We analyzed the Na^+^ and K^+^ conductances of AtHKT1;1 in their native root stelar cells, showing that AtHKT1;1 shows clear Na^+^ currents but AtHKT1;1-dependent K^+^ currents were not detected (Figures 3 and 5). These findings are in line with analyses of AtHKT1;1 in *Xenopus* oocytes and yeast, which showed a Na^+^ selectivity of this transporter [10], [11]. AtHKT1;1-mediated currents in root stelar cells also show no strong voltage dependence, consistent with previous findings in *Xenopus* oocytes [10], [11].

A recent study expressing the rice HKT transporters OsHKT2;1 and OsHKT2;2 in plant cells and tracer flux and ion accumulation analyses showed transport properties of these HKT transporters in plant cells [47] comparable to those found in heterologous expression systems [11], [25]. Previous electrophysiological studies in *Xenopus* oocytes have shown that HKT transporters can carry large outward currents upon depolarization of the plasma membrane [10], [20], [25], [45], [47], [60], [61], [62], [63], [64]. In these studies the cytoplasmic solution of oocytes is not controlled and the main intracellular cation is likely to be K^+^ mixed with Na^+^ emanating from HKT-mediated Na^+^ uptake during oocyte culture and therefore the question, whether outward currents can be carried by Na^+^ or other ions has remained unknown. In the present study, whole-cell recordings were performed with NaCl or KCl in the pipette solution that equilibrated with the cytoplasm of root stelar cells. These experiments clearly showed that AtHKT1;1 could effectively transport Na^+^ efflux from cells upon membrane depolarization (Figure 3).

However, our data also indicate an unexpected role for extracellular Na^+^ in gating and enhancing AtHKT1;1 activity (Figure 2A and B). When AtHKT1;1 was expressed alone in *Xenopus* oocytes a large reduction in outward currents upon removing extracellular Na^+^ was observed even though the driving force for AtHKT1;1-mediated efflux was greatly enhanced (Figure 4). Our present findings indicate a possible additional regulation of AtHKT1;1 activity via direct and/or post translational mechanisms. A related study of Møller et al. [23] was conducted in the *Arabidopsis* C24 background while the present study was conducted on Columbia background, and different conditions may affect post-translational regulation of AtHKT1;1 activity. For example, a recent study showed differential Na^+^ accumulation and differential regulation of Na^+^ transporter expression, including *AtHKT1;1*, in the *Arabidopsis* Columbia and C24 backgrounds [65]. Thus the present study indicates that Na^+^ may regulate AtHKT1;1 activity such that activity is largest when Na^+^ levels rise in the xylem sap.

The present data comparing wild-type and *athkt1;1* show that AtHKT1;1 can transport Na^+^ currents into root stelar cells as well as outward Na^+^ currents in plant cells, with the direction of Na^+^ transport being controlled by the membrane potential and the prevailing ionic conditions. Uptake of Na^+^ via AtHKT1;1 into xylem parenchyma cells is clearly supported by previous xylem sap ion concentration analyses of *athkt1;1* mutants [17], [18], [19]. Whether AtHKT1;1 mediates substantial Na^+^ efflux into the xylem sap (xylem loading) has not yet been analyzed *in vivo*. For example, previous research has measured a xylem sap Na^+^ concentration of 0.3 mM Na^+^ in non-stressed plants and about 5 mM Na^+^ for salt-stressed plants that were exposed to 75 mM NaCl for 2 days [17]. For non-stressed plants, assuming a cytoplasmic concentration of 3 mM Na^+^ in xylem parenchyma cells, depolarizations positive of −58 mV would result in AtHKT1;1-mediated Na^+^ loading into the xylem at 20°C. Activation of anion channels could produce such depolarizations [55], [66], [67]. Nevertheless, the question whether type 1 HKT transporters can mediate net release of Na^+^ into the xylem sap will require further analyses, in particular in reference to the reduction in AtHKT1;1-dependent outward currents when extracellular Na^+^ is removed, as found here (Figures 2A, B and Figure 4).

AtHKT1;1 in *Arabidopsis* and OsHKT1;5 in rice, not only reduce Na^+^ accumulation in the xylem sap, but also increase K^+^ concentrations in xylem sap and in leaves [17], [20]. In the present study, whole-cell K^+^ currents with K^+^ as the major cation dialyzing the cytoplasm of root stelar cells were indistinguishable between wild-type and *athkt1;1* mutant plants (Figure 5). These data show that K^+^ is not substantially transported by AtHKT1;1, as K^+^ currents in root stelar cells of wild-type plants would have otherwise been predicted to be measurably larger than those in *athkt1;1* mutant plants, which was not the case. An alternate explanation of these observations could be that AtHKT1;1 was down-regulated under these conditions in wild-type xylem parenchyma cells. Further research is needed to derive a precise permeability ratio for Na^+^ vs. K^+^ selectivity. Previous research has shown that when AtHKT1;1-mediated currents were recorded in the presence of Na^+^, shifting of the extracellular K^+^ concentration did not significantly shift the reversal potential of AtHKT1;1-mediated currents, demonstrating the Na^+^ over K^+^ selectivity of AtHKT1;1 [10].

Data suggesting that AtHKT1;1 does not mediate substantial K^+^ transport in native root stelar cells, is consistent with the observation that knockout of *AtHKT1;1* affected neither root influx nor root-to-shoot transfer of ^86^Rb^+^ (K^+^) [19], [68]. Thus, the HKT-mediated increase in K^+^ concentrations in wild-type xylem sap and leaves [17], [20] is likely to be an indirect effect of AtHKT1;1, as proposed previously [12], [17]: Membrane depolarization caused by Na^+^ influx into xylem parenchyma cells via AtHKT1;1- and OsHKT1;5-catalyzed transport will depolarize these cells. Such depolarization both activates outward-conducting K^+^ efflux channels and provides a driving force for K^+^ release (Figure 5) [54], [55], [56], [57], [58].

Several studies have suggested that HKT transporters could mediate passive ion channel-like transport [35], [45], [46], [47]. However, previous studies have not controlled the cytoplasmic solution during electrophysiological recordings and reversal potentials of HKT transporter-mediated currents in *Xenopus* oocytes usually do not show clear Nernstian shifts, with the caveat that the intracellular cation concentrations are not easily predictable and likely to be highly variable due to HKT transporter-mediated cation influx as soon as these transporters are functionally expressed during oocyte post-injection incubation periods. Patch clamp analyses of AtHKT1;1-mediated Na^+^ currents in root stelar cells at clamped cytosolic ion concentrations show clear Nernstian reversal potential changes for Na^+^ (Figure 3), showing that AtHKT1;1 has a passive biophysical ion channel-type transport activity. Note that a Na^+^-selective uniporter would be equivalent theoretically and experimentally to an ion channel for reversal potential calculations (see equations in: [61], [69]).

Thus the properties of AtHKT1;1 in native root stelar cells correlate well with the AtHKT1;1 properties analyzed in heterologous expression systems. Moreover, whole-cell patch clamp analyses of AtHKT1;1-dependent Na^+^ currents in root stelar protoplasts with defined intracellular ion concentrations demonstrate Nernstian channel transport properties of AtHKT1;1. The above advances point to interesting new open questions regarding AtHKT1;1 and HKT transporters in general, as well. The ability to directly analyze electrophysiological properties of an HKT transporter in its native cell system in wild-type and genetic mutants provides a powerful avenue to address outstanding questions in salt tolerance mechanisms.

## Materials and Methods

### Plant materials

An *Arabidopsis thaliana* control line, E2586, that expresses GFP in stelar cells was used in the present study [23], [52], [53]. This enhancer trap line was crossed into the *athkt1;1-4* loss-of-function mutant background and homozygous *athkt1;1-4* X E2586 seeds were isolated (Columbia-0 background).

### Growth conditions

Seeds were surface sterilized using 50% bleach with 0.1% Triton X-100 for 5 min and rinsed with sterile de-ionized water five times, and placed on 10-cm-wide Petri dishes with half strength Murashige and Skoog medium, 1% Sucrose, 5 mM MES (2-(N-Morpholino)-ethanesulfonic acid), 0.8% photo agar, pH adjusted to 5.6 with KOH. Seeds were vernalized for 2 days at 4°C and then transferred to a growth chamber with a temperature of 21°C, an approximate photon flux density of 100 mmol m^−2^ s^−1^, and a 16-h photoperiod.

### Semi-quantitative RT-PCR assays

To analyze the expression levels of AtHKT1;1 in E2586 and *athkt1;1-4*×E2586 lines, semi-quantitative RT-PCR analyses were performed. Total RNA samples were isolated from root tissues of 2-week-old seedlings using the RNeasy Plant Mini Kit (Qiagen) followed by DNase digestion and RNA purification, and then first-strand cDNA was reverse transcribed with a first-strand cDNA synthesis kit (GE Healthcare) at 37°C for 1 h. The AtHKT1;1 transcript was amplified using the gene-specific primers forward primer 5′-CCACATGGACAGAGTGGTGGCAAAAATA-3′ and reverse primer 5′- TTAGGAAGACGAGGGGTAAAGAATCC-3′. Amplification of the ACTIN2 (AT3G18780) (forward, 5′-CTAAGCTCTCAAGATCAAAGGCTTA-3′; and reverse, 5′-ACCTTGAAACTCTTTGCAATGTTAA-3′) mRNA was used as an internal control.

### Isolation of protoplasts

9- to 13-day-old roots were used for protoplast isolation. Minor modifications were made to a method described earlier [70]. In brief, roots were chopped into small pieces in 3 ml enzyme solution consisting of 1.5% Cellulase Onozuka RS (Yakult Honsha, Tokyo), 1% Cellulysin (CalBiochem, Nottingham, UK), 0.1% Pectolyase (Sigma), 0.1% Bovine Serum Albumin, 10 mM KCl, 10 mM CaCl_2_, 2 mM MgCl_2_, 2 mM MES, 200 mM D-mannitol, pH 5.6 adjusted with Tris. Roots were gently shaken at 60 rpm in the enzyme solution at 26°C for 3 h. Protoplasts were filtered through a 35 µm nylon mesh, washed with wash solution (1 mM KCl, 1 mM CaCl_2_, 1 mM MgCl_2_, 5 mM Sucrose, 2 mM MES, pH 5.6 adjusted with Tris, osmolality was adjusted to 290–300 mmol·Kg^−1^ with D-mannitol) and collected by centrifugation for 7 min at 150 g. Isolated protoplasts were stored on ice for experiments and used on the same day. Protoplasts with diameters between 20 and 25 µm were used in all patch-clamp experiments reported here. For calculation of average current densities, we used an average of protoplast diameter of 23 µm.

### Electrophysiology

Whole-cell patch-clamp experiments were performed as described previously [71], [72]. Fluorescent root stelar protoplasts were voltage-clamped using an Axopatch 200 amplifier (Axon Instruments). Glass pipettes with size of 1.5–1.8×100 mm (Kimble Chase) were pulled using a multistage programmable puller (Model P-87, Sutter Instruments). The pipette resistance filled with a 50 mM NaCl or KCl containing pipette solution was about 10 MΩ. To estimate the diffusional equilibrium time between the pipette and protoplast, equations described by Pusch and Neher [73] were used and the diffusion time constant was approximated to less than 30 seconds for Na^+^ and K^+^ given the protoplast diameters of up to ∼25 µm. During patch clamp experiments, currents were recorded 7–10 minutes after access to the whole-cell configuration. The bath solution contained 0, 50 mM NaCl or 50 mM KCl, 2 mM CaCl_2_, 5 mM MES, pH 5.7 adjusted with Tris and the pipette solution contained 50 mM or 5 mM NaCl or 5 mM KCl, 1.3 mM CaCl_2_, 3 mM EGTA, 5 mM HEPES, pH 7.2 adjusted with Tris. The osmolalities of the bath and pipette solutions were adjusted to 290–300 mmol·Kg^−1^ with D-mannitol. An Ag-AgCl/1 M KCl-agar bridge was used as a reference bath electrode. Ion activities were corrected using previously described equations [74]. Liquid junction potential was measured using Clampex 10. Protoplasts were perfused with bath solution during current recordings with the bath electrode tip located at the perfusion tube exiting the bath solution to avoid KCl diffusion into the bath. Experiments were performed at room temperature (23°C). All whole-cell recordings with an initial seal resistance of more than 5 GΩ and stable whole-cell recording were used for data analysis. Data were acquired using voltage ramp protocols, with the membrane voltage being ramped from +99 mV to −141 mV over 1 s, and the holding potential between ramps (V_H_) was −1.5 mV. Data were filtered at 1 kHz and stored on a computer through an Axon digidata 1440A data acquisition system (Molecular Devices) and analyzed with Axon Clampfit 10 (Molecular Devices), Microsoft Excel and Origin 8.0 (Originlab Corporation).

### Heterologous Expression in *Xenopus* Oocytes

The *AtHKT1;1* cDNA (Columbia ecotype) was isolated and cloned (AT4G10310) using the *AtHKT1;1* sequence from the Landsberg erecta ecotype [10]. Capped cRNA of *AtHKT1;1* was transcribed from a linearized plasmid construct pNB1::*AtHKT1;1* with the mMESSAGE mMACHINE in vitro transcription kit (Ambion, Austin, TX, USA). 20 ng of *AtHKT1;1* cRNA was injected into *X. laevis* oocytes, while an equivalent volume of water was injected into oocytes as controls. Oocytes were kept for 1 to 2 days at 18°C in K^+^ ringer solution composed of 115 mM KCl, 1.8 mM CaCl_2_, 1 mM MgCl_2_, 10 mM HEPES-Tris, pH 7.4. Two-electrode voltage clamp experiments were performed using a Dagan TEV-200 amplifier (Dagan Corporation, Minneapolis, MN, USA). Oocytes were perfused with solutions containing 1 mM CaCl_2_, 1 mM MgCl_2_, 10 mM HEPES, 200 mM D-Sorbitol, pH 7.4, and the indicated concentrations of NaCl. Clampex 10 (Axon Instrument, CA, USA) was used for electrophysiological measurements. Voltage steps were generated from −150 mV to +110 mV in +20 mV increments. Microelectrodes were filled with 3 M KCl. A 3 M KCl agar bridge was used as bath electrode. All experiments were performed at room temperature (23°C).
